# Liver in the Chest: A Case of a Large Traumatic Diaphragmatic Rupture

**DOI:** 10.7759/cureus.17028

**Published:** 2021-08-09

**Authors:** Rebecca Innes, Mohan Kulkarni

**Affiliations:** 1 Surgery, Henry Ford Health System, Jackson, USA; 2 Thoracic Surgery, Henry Ford Health System, Jackson, USA

**Keywords:** trauma, major trauma, diaphragm injury, thoracotomy, motor vehicle accident, liver herniation, blunt trauma

## Abstract

Motor vehicle collisions (MVC) cause more than one million deaths annually and an estimated 20-50 million significant injuries. They can cause blunt and penetrating trauma. Blunt diaphragmatic rupture is generally associated with multiple severe injuries due to the high force needed to cause the injury. Traumatic diaphragmatic rupture (TDR) is normally identified during advance trauma life support (ATLS) secondary survey, after other more serious injuries are identified in the primary survey. We present a case of a patient who was involved in a MVC with multiple injuries, which were treated appropriately, prior to identification and treatment of a severe right-sided diaphragm injury. Imaging showed only a persistent right hemidiaphragm elevation. Intra-operative findings consisted of complete herniation of the liver with a Grade IV, 30 cm, right-sided diaphragmatic rupture. The herniated liver was repositioned and the diaphragm primarily repaired without complication. This case highlights a severe injury from a blunt MVC and rapid successful recovery of the patient once appropriately treated.

## Introduction

Blunt traumatic diaphragmatic rupture (TDR) is caused by high energy damage force and is associated with multiple life-threatening injuries [1]. TDR is more commonly diagnosed on the left side (80%), likely attributed to the liver protecting the right side of the diaphragm (20%). The management of the patient is done according to advance trauma life support (ATLS) protocol. Injuries are frequently missed initially due to the severity of concomitant injuries that are often treated first [2]. Chest films are misinterpreted as showing hemi-diaphragm elevation, hemothorax, pneumothorax, or sub pulmonary hematoma. The only indication of right-sided injury may be right diaphragm elevation on a chest X-ray. A high index of suspicion of injury to the diaphragm is needed to reduce the morbidity associated with a delayed or missed injury [3,4].

## Case presentation

A 24-year-old male presented to Level 2 trauma center, via emergency medical services (EMS) after a motor vehicle accident. The patient was a restrained passenger involved in motor vehicle collision (MVC) in which air bags were deployed. Per EMS, there was a significant intrusion on the passenger side of the vehicle with a prolonged extrication time. The patient reported pain in his right arm, right chest, and right pelvis. The patient had no significant past medical or surgical history. 

On initial trauma code physical exam, the patient was tachycardic with a heart rate of 119 beats per minute and his systolic blood pressure remained above 100 mm of Hg. On chest auscultation, the right lung was clear while the left side demonstrated crackles. The pelvis was stable, but there was tenderness to the right pelvis with the movement of the right leg. The right shoulder was noted to have a significant deformity and an associated open humeral fracture. Significant laboratory work demonstrated a leukocytosis of 22,100 cells/µL, hemoglobin of 11.2 g/dL, and liver enzyme elevation of ALT 638 IU/L, AST 394 IU/L. Chest radiography (CXR) showed a moderate right layering pleural effusion and a possible hemothorax. Right shoulder radiography demonstrated a severely comminuted, angulated, and impacted right humeral neck fracture. Pelvis radiography demonstrated acute displaced fractures involving the right superior and inferior rami. Focused Assessment with sonography for trauma (FAST) exam was negative. Computed tomography (CT) of the chest, abdomen and pelvis demonstrated the following: a right-sided traumatic hemothorax, a small right-sided traumatic pneumothorax with severe right-sided pulmonary contusion; a Grade 3 right renal laceration; a Grade 4 liver laceration without evidence of active arterial extravasation; multiple fractures including the right humerus, right fourth rib, inferior sternum, right S1 hemisacrum, S4 sacral segment and right superior and inferior pubic rami. 

**Figure 1:**
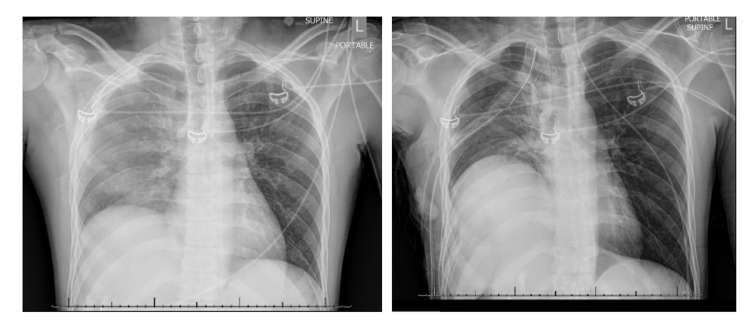
Chest X-ray before (left) and after (right) tube thoracostomy placement with right-sided diaphragm elevation.

The patient was immediately treated with right humeral reduction and splinting for the open fracture. A right-sided thoracostomy tube was placed for the hemo/pneumothorax with 500 ml of blood initially evacuated. The patient was admitted to the Surgical Intensive Care Unit and transfused two units of packed red blood cells. The patient was taken to the operating room (OR) with Orthopedic surgery for open reduction, internal fixation of the right humerus and pelvic fractures. Postoperatively, on routine CXR comparison, it was noted that the patient had persistent elevation of the right hemi-diaphragm since tube thoracostomy placement (Figure 1). Thoracic surgery was consulted for further evaluation.

**Figure 2:**
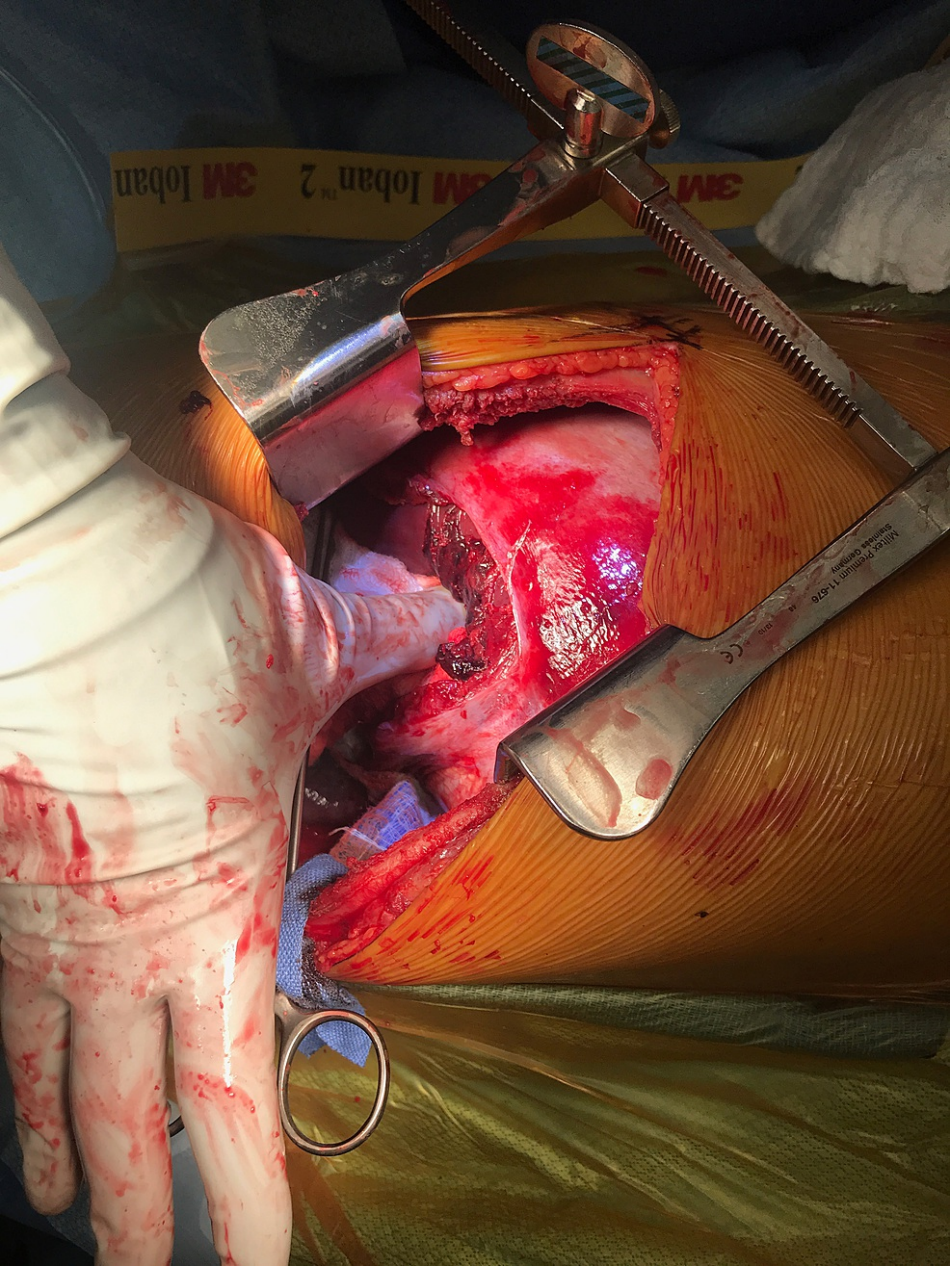
Intra-operative photo of the liver reduced back into abdominal cavity through large diaphragm defect.

The patient underwent a video-assisted thoracoscopy on suspicion of a diaphragmatic injury. It was noted upon entry into the thoracic cavity, that the liver was almost completely herniated into the chest. The chest was then opened via a right posterolateral thoracotomy through the 7th intercostal space. After dissection down to the level of the pleura, the liver was carefully reduced back into the abdominal cavity (Figure 2). 

**Figure 3:**
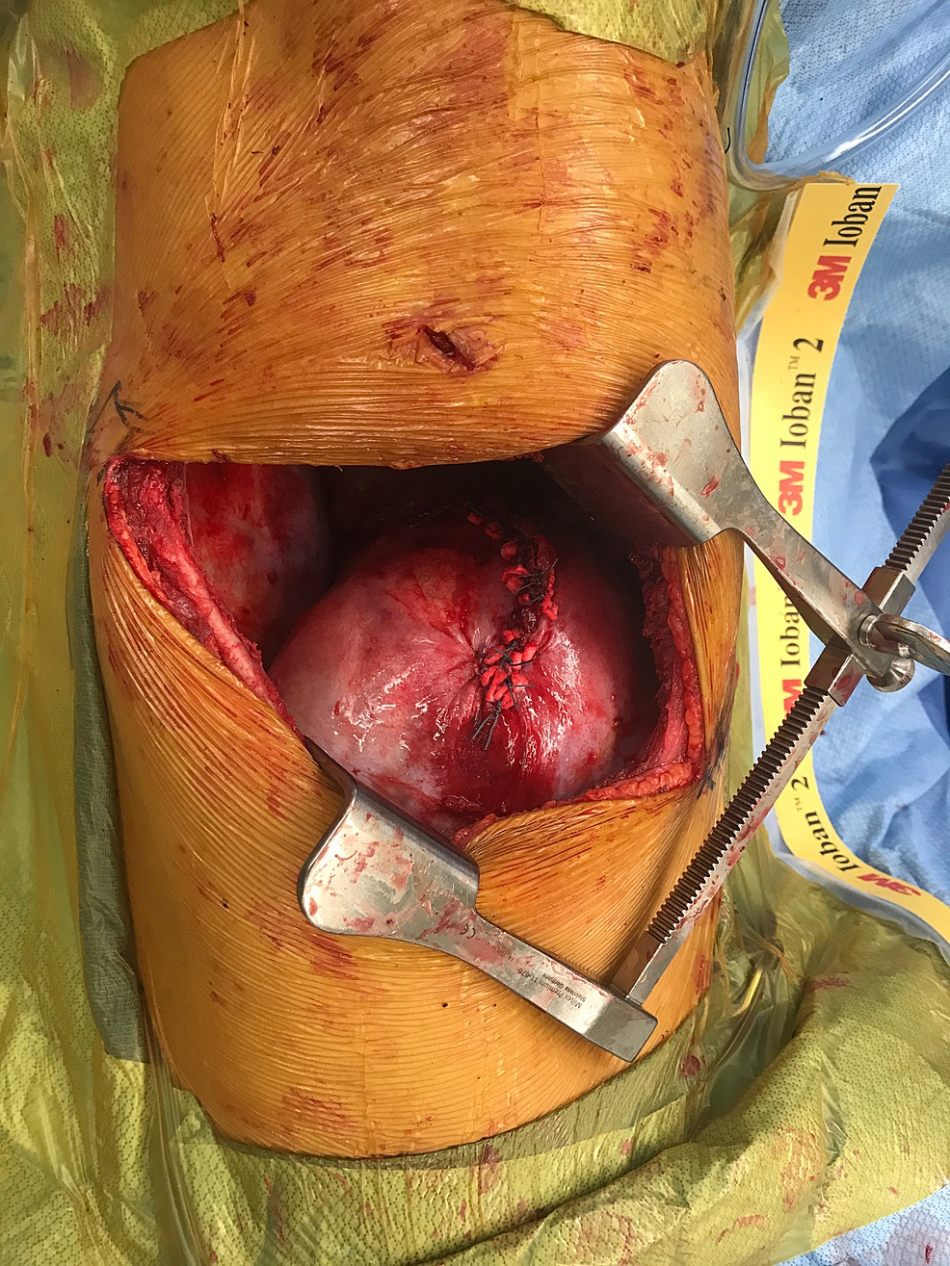
Completed primary repair of diaphragm injury.

The entire length of the diaphragmatic injury was noted to be 30 cm with the most medial aspect torn approximately 5 mm from the inferior vena cava (IVC). The phrenic nerve appeared to be involved in the tear near the IVC. The edges of the torn diaphragm were closed primarily using interrupted Ethibond pledgeted sutures (Figure 3).

Two thoracostomy tubes were secured. The chest wall was then closed in layers. The patient remained hemodynamically stable throughout the procedure. 

The patient’s post-operative course was uncomplicated. The thoracostomy tubes were both removed on the third post-operative day. The patient was evaluated throughout the hospitalization by physical and occupational therapy services with recommendations to continue care at a short-term rehab facility upon discharge. The patient continued to clinically improved and was discharged to the rehab facility on post-operative day 5. 

The patient was followed up after three weeks in the thoracic surgery outpatient clinic. He reported feeling well at the visit with only mild chest pains that intermittently radiated to his right shoulder. A follow-up CXR demonstrated a small right-sided pleural effusion, but no other abnormalities. The patient was deemed recovered at this time and was instructed to follow up as need. 

## Discussion

TDR is estimated to occur in approximately 1%-7% of patients with major blunt trauma [1]. In a year review of 2012 by the National Trauma Data bank, 3873 out of 833,309 patients had a traumatic diaphragm injury (46%). Of those, 1,240 (33%) patients had a blunt mechanism and 2,543 (67%) had a penetrating mechanism [5]. This is similar to other case series estimating injury from 0.8 to 8%. Diaphragmatic injury with associated hernia of abdominal contents may result in compromised cardiopulmonary function due to a displaced mediastinum, impaired venous return from a displaced liver or pressure on the inferior vena cava, and decreased pulmonary function of the affected side [6]. Long-term sequelae of injury that is not noted on index hospitalization can result in herniation, obstruction, incarceration or strangulation of intra-abdominal contents as well [7]. Minimally invasive procedures such as diagnostic thoracoscopy and laparoscopy are used to diagnose TDR, as long as the patient is hemodynamically stable. These procedures are beneficial where imaging findings do not have a clear diagnosis. Larger diaphragmatic defects often lead to the procedure conversion to an open thoracotomy or laparotomy. Multiple literature reviews do not demonstrate an optimal surgical approach for repair and the decision on surgical approach appears to be largely patient dependent and selected on a case-by-case basis [8].

Our patient was taken to the operating room for high clinical suspicion of blunt diaphragmatic injury due to persistent CXRs showing right hemi-diaphragm elevation although injury was not present on CT scan. The surgical approach chosen was a laparoscopic thoracoscopy ultimately converted to open thoracotomy after a large right-sided diaphragm injury with liver herniation was found. This injury had the potential to be catastrophic, as the significant tear in the diaphragm was noted to be 5 mm from the inferior vena cava. Due to no tissue loss or tension on the diaphragmatic edges, a primary repair was easily performed, and a patch or mesh was not needed. This was largely attributed to prompt diagnosis and treatment. The injury noted was a Grade IV injury based on the Organ Injury Scale with a 30cm laceration, and had the potential to develop into a Grade V if the diagnosis had been further delayed [9]. The outcome was good for this patient, as they no longer required follow-up two months after the index operation, and the patient continued to work with physical therapy for orthopedic injuries sustained.

## Conclusions

In conclusion, blunt traumatic diaphragmatic rupture is a serious injury that can lead to significant complications if left untreated. It is difficult to diagnose with imaging studies alone. The diagnosis often depends on the providers clinical suspicion of injury, and prompt treatment must then ensue. Surgical therapies for diaphragmatic rupture are employed for diagnosis and treatment, and in our case lead to rapid recovery once treated appropriately. This case study demonstrates the success of prompt treatment of a severe blunt right-sided diaphragmatic injury with liver herniation and a rapid recovery after surgery with minimal morbidity.
